# “The Important and Unfamiliar”: Richard Hofstadter’s *The American Political Tradition*

**DOI:** 10.1007/s12115-018-0225-2

**Published:** 2018-02-23

**Authors:** Andrew Snodgrass

**Affiliations:** Jesus College, Cambridge, Great Britain CB5 8BL UK

**Keywords:** Consensus, Historiography, Liberalism, Pluralism

## Abstract

This paper addresses the continued association of Richard Hofstadter with consensus history. More specifically, it challenges the view that the origins of this conservative trend in American history can be located within *The American Political Tradition*. Whilst primarily concerned with reinterpreting Hofstadter’s work within its original context, the paper raises questions regarding author intention and both the reception and shifting perceptions of works of history.

Shortly after the publication of *The American Political Tradition*, Richard Hofstadter received a castigatory letter from an enraged reader. Amidst the insults and accusations, the correspondent declared the work a “rotten, disloyal book.” To the reader, Hofstadter’s hatred of his native land seemed apparent and his “filthy lies” were clearly part of the wider assault on America that had been launched by the New Deal.^1^ That the book elicited such an angry response due to its critical tenor might seem perplexing given Hofstadter’s later, and continued, association with consensus history. Indeed, the book is often considered to be the foundational text of the consensus school, a historiographical movement criticised for its “strikingly conservative” vision of the American past.^2^ In order to explain these remarkably divergent assessments and the shift in perception over time, it is necessary to reconsider both the book and the history of the concept of consensus history.

An early draft of Hofstadter’s introduction, written after completion of the essays, gives a remarkable insight into his original intention for the work. He indicated that his primary aim was not to search for a broad interpretation of the American political tradition, but rather to examine the thought and character of a selection of the nation’s most influential political leaders. In doing so, he wished to dispense with the familiar interpretations in favour of portraits that brought the neglected aspects of the nation’s past to the fore**.** It was not a typical work of political portraiture, and he did not intend to produce exhaustive biographical accounts of his subjects. Instead, he saw his sketches as those of the historical caricaturist, the essays marked by the deliberate exaggeration of those features he deemed salient. If he were to choose a unifying theme, it was simply a desire to search out “the important and unfamiliar.”^3^

What Hofstadter deemed to be important was, of course, reflective of his own intellectual and political position in the middle years of the 1940s. Although published towards the end of that decade, Hofstadter commenced work on the book in 1943 and later described the work as a product of the ideological debates and social criticism of the 1930s. During his student years, radical politics had played a pivotal role in his life and had been central to his intellectual formation. Although his time as an active member of the Communist Party had been brief, his estrangement had been primarily due to his discomfort at the intellectual sterility and rigidity of thought within the party. As he explained after his decision to leave, “I hate capitalism and everything that goes with it. But I also hate the simpering dogmatic religious-minded Janizaries that make up the CP.” His time within the party served to cast doubt on much of what he had accepted as certitudes and his disillusionment with Communism was swiftly followed by a disavowal of his Marxism. Nevertheless, his withdrawal from active politics did not lead to willing acceptance of the status quo, but rather to a feeling of political detachment. In a letter to his brother-in-law in which he spoke of the personal anguish of his sense of alienation, he mournfully concluded, “We are the people with no place to go.”^4^

Hofstadter’s final years as a graduate student were ones of political and intellectual uncertainty. The successful completion of his doctoral thesis in 1942 and, later that year, a first academic post at the University of Maryland went some way to ease his concerns about the future. Although he found little joy in his teaching role there, he relished the intellectual and social camaraderie provided by a small group of colleagues who shared many of his political concerns. He wrote enthusiastically of lunchtime discussions in which he and his friends would “sit around and tear sandwiches and bitterly denounce Churchill, FD, the State Dep., the military, southerners, and all aspects of the status quo.”^5^ Despite his feeling ill at ease within the staid and conservative atmosphere of wartime Maryland, this small fraternity of radical spirits provided an intellectual oasis for Hofstadter.

Hofstadter’s closest friend within the group was a recent appointee in the sociology department, C. Wright Mills. The two had felt an immediate sense of intellectual kinship and had formed a close friendship. That their views diverged in later years has led them to be cast as opposites, the conservative historian of consensus and the radical mentor of the New Left. However, this distorted caricature of the two men as polar types fails to take account of the commonality of their thought and shared intellectual concerns, particularly strong at the outset of their careers. Whilst the two were at Maryland there was considerable agreement on political issues, as both men made their critical observations from what Hofstadter described as the most “thorough-going leftist pt. of view.”^6^ Despite their differing paths, Hofstadter and Mills met a time when their political trajectories were intersecting and the influence of their friendship on their academic work was inevitable.

The continued radicalism, albeit outside active politics, of Hofstadter’s years at Maryland provide an essential backdrop to the essays contained within *The American Political Tradition*. It was a work conceived from a vantage point well to the left, and reflective of Hofstadter’s sense of detachment from mainstream politics. His distaste for the political system was writ large in his draft introduction. Hofstadter saw American political history as a story of competing dramas in which the lead actors were those politicians, often dynamic and personally appealing, whose performances captured the hearts of the nation. As with a theatrical production, successful political campaigns were thoughtfully staged, carefully timed and always sensitive to the tastes and prejudices of the public. The debates over policies were adjudged to be mere gestures, created to give the illusion of reality. It was an illusion that entranced not only the majority of the voting public but, to Hofstadter’s dismay, most historians. They had too readily succumbed to the spell of the drama and too often accepted the “dramatic values intended by the authors of the script, transfer[red] the fictions of the stage to the printed page, and hand[ed] them to posterity.”^7^

In contrast, Hofstadter saw his own writing as that of one who watched the political drama from a position “in the wings”. From there, he could observe the actors behind the characters and analyse the inner workings of the production rather than simply view the performance on stage. His essays would tear away the masks behind which the historical actors hid themselves from public view. The resultant work was one that was intensely critical and unsparing in its assessment of the nation’s political leaders. It was, in the words of C. Van Woodward, “a book without a hero.”^8^

As the book was going through its final edit, Hofstadter began to worry about the reaction to what he described as his “disgruntled, critical, alienated tone.” In a letter to his mentor, Merle Curti, he confided, “I had the necessary courage to write it but I am now beginning to wonder if I have the courage to see it through publication.” He was certain that the tenor of the essays would draw significant criticism and was prepared for an “extremely poor reception”.^9^ Given these concerns, he was unlikely to have been surprised by the fact that an enraged reader felt compelled to vent their ire after reading the book. Interestingly, he had expected the greatest criticism from those within the historical profession and there were some amongst the initial reviewers who felt uneasy at Hofstadter’s iconoclastic style. There was a feeling that the overriding desire to debunk had led to the choice of materials being made for effect rather than in pursuit of historical balance. Yet, the irreverence of the portraits and the ease and grace with which he dismantled the myths of the nation’s leaders drew more praise than disapproval. As one reviewer summed it up, the book was “a fresh breeze ventilating the stodgy atmosphere of academic research.”^10^ Whilst opinions differed on the tone of the work and the style of his writing, there was universal agreement that Hofstadter’s study was one that was intensely critical of the nation’s political leaders.

Hofstadter had set out to challenge both historical orthodoxy and popular mythology, and he was uncompromising in his judgments. His chapter on Thomas Jefferson was reflective of the mordancy with which he set about the task. Of Jefferson, he observed that the “mythology…is as massive and imposing as any in American history.” Despite abundant scholarship that deflated the roseate image of Jefferson, much of which Hofstadter consulted in his study, the myth persisted. As Hofstadter remarked scathingly, “no aristocrat… could be quite the democrat Jefferson imagined himself.” Yet, to Hofstadter’s dismay, the popular characterization of Jefferson as a crusading democrat retained its force. Likewise, “the Lincoln legend has come to have a hold on the American imagination that defies comparison with anything else in political mythology.” In the case of Lincoln, “the greatest character since Christ”, “the first author of the legend…was Lincoln himself.” Lincoln had been fully aware of his role as an exemplar of the possibilities for the simplest of men and he ensured he performed it masterfully.^11^

The mythology that Hofstadter seemed most keen to deflate was that surrounding Franklin D. Roosevelt. His earlier work on the sharecroppers had been a critical assessment of the darker political reality that lay behind Roosevelt’s New Deal initiatives, and his assessment in *The American Political Tradition* was, in many ways, informed by the same radical impulse. In his student days Hofstadter had liked to amuse his friends with sardonic parodies of Roosevelt, and he retained much of his contempt for the revered president. Hofstadter bemoaned the fact that “Roosevelt is bound to be the dominant figure in the mythology of any resurgent liberalism.” It was undoubted that “there were ample texts for men of good will to feed upon,” but he urged caution in putting faith in “the wonder-working powers of the great man.” Roosevelt’s significant appeal masked the emptiness of his political philosophy. The Roosevelt myth seemed to Hofstadter to be the most dangerous to the project of revitalizing the liberal-left.^12^

Throughout the essays, Hofstadter was keen to remind his readers of the incompatibility of the virtues assigned these mythic figures with the dirty work of politics. Jefferson is described as “too successful a politician to be the crusading democrat of legend.” Lincoln was “completely the politician by preference and training” who had learned “the deliberate and responsible opportunism” necessary for success. He is portrayed as a man whose ideas and beliefs remained secondary to political strategy. Indeed, his success in 1860 entitled him “to a place among the world’s great political propagandists.” For those who had heralded Roosevelt as the great liberal saviour, Hofstadter counselled them to consider that his turn to the left had been motivated solely by political gain. When one scraped beneath the surface of the myths surrounding politicians one always found the murky underbelly of political motivation.^13^

As Hofstadter lifted the masks from the nation’s leaders he was struck by the clear discrepancies between their pronouncements and their practices. He wrote of Jefferson that he must “be measured in whole, not in part, in action as well as thought.” Despite his Enlightenment ideas, he “was not in the habit of breaking lances trying to fulfil them.” The presidency of Theodore Roosevelt was characterized by “a hundred times more noise than accomplishment.” Woodrow Wilson was forced to “turn his back on his deepest values,” as he led the nation into World War One. F.D.R.’s failure to purge his party of its conservative elements was symbolic of “the political bankruptcy of the New Deal.” Hofstadter presented the nation’s political heroes as men inhabiting a moral and intellectual twilight, masters of manipulation and deception, cloaked beneath a charade of the highest integrity. Indeed, Wendell Phillips was the only figure within the book who refused to compromise his ideals. Instead he preferred to keep his eye on the “ultimate potentialities”, irrespective of the restraints, or personal cost. In the one positive portrayal in the book, we find a man unencumbered by political office, and prepared to present himself to the public naked and unmasked.^14^

Despite the distinctly critical temper of the book, it has come to be seen as symbolic of a shift in Hofstadter’s political and intellectual viewpoint, a work indicative of his move from radicalism to the pluralist centre. One significant reason for the shifting perception is the focus on the book’s introduction. It was his opening remarks that would draw later critics to locate the origins of consensus history in *The American Political Tradition*. However, as several of the initial reviews noted, it did not seem to be completely integrated with the rest of the study. Daniel Aaron suggested that whilst “it is difficult to quarrel with Mr Hofstadter's personal estimates of the men he is presenting…the implications of his thesis, advanced rather obliquely in his introduction, are not completely clear.”^15^ Arthur Schlesinger Jr. considered the introduction to be “perfunctory” and felt that the essays contained within the book rendered it “not false, but somewhat irrelevant.”^16^ It is indeed an irony that the element of the book that contemporary reviewers felt was not quite reflective of the work as a whole became the cornerstone of later interpretations.

The apparent incongruity of the book’s title and introduction, and the main text, was clarified by Hofstadter in a preface to the 1967 Hebrew edition of the work. Hofstadter explained that Knopf had been concerned that the work read as a collection of unrelated parts and requested that he write a unifying introduction. Despite having no thought of developing a theory of American politics, the editors convinced him of the need to stress the value of the book as an important re-interpretation of the American past. Indeed, it is clear from the correspondence with Knopf that they viewed the opening statement as key to the commercial success of the book. At Knopf’s request, his original introduction was re-written and, despite his sense of unease, Hofstadter produced a statement that indicated a degree of intellectual completeness that he had wished to avoid. The issue of the title proved equally contentious, as Hofstadter was persuaded to drop his rather modest and more fitting title, *Men and Ideas in American Politics*, for something considered to have greater saleability. As books of essays were considered commercially unviable, the title had to indicate a coherence and ambition that would arouse the interest of the reading public. As Arthur Schlesinger Jr. had so perceptively discerned, the title and introduction were, to a great degree, “tacked on at the last moment.”^17^

The opening remarks have, in many ways, overshadowed the essays themselves. His statement that “above and beyond temporary and local conflicts there has been a common ground, a unity of culture and political tradition, upon which American civilization has stood,” was one that proved controversial.^18^ His assertion that American political life was better viewed as a story of shared assumptions than of ideological struggles was a clear rebuttal of the work the Progressive generation. Hofstadter felt that the emphasis on conflict had run its course and his work offered a necessary corrective. In a challenge to the Progressive orthodoxy, he sought to address the underlying premises upon which the nation had existed. The emphasis placed by Hofstadter’s on ideological agreement rather than conflict was undoubtedly a political statement. As such, it was one that one that would inevitably be reinterpreted as the political landscape shifted in later years.

It was not until the early 1960s that critics, no doubt influenced by images of formerly radical intellectuals retreating into quietude, began to describe Hofstadter as a consensus historian. The term was originally coined by John Higham in his 1959 essay, “The Cult of ‘American Consensus’: Homogenizing Our History.” Higham set out to summarize what he saw as the growing tendency towards conservatism in American history. It is important to note that Hofstadter is mentioned by Higham only in passing and *The American Political Tradition* not at all. The key works were those of Louis Hartz and Daniel Boorstin. Hartz’s *The Liberal Tradition in America* represented the positive impact of the revolt against the dualism of the Progressives, his work a critical account of the dominance of Lockean thought in American political history. Boorstin, on the other hand, was responsible for “a drastic revision of American history in a conservative direction.” *The Genius of American Politics* took consensus history to its most extreme, eliminating all aspects of conflict from the American past and denying the impact of ideological systems of thought. This “bland approval of American institutions” was symptomatic of the deadening effect of contemporary conservatism on the writing of history. Higham’s concept of consensus immediately struck a chord and swiftly entered the discourse of American historiography. Indeed, much like Hofstadter’s introduction, its influence far exceeded the initial intentions of the author.^19^

In an address to the 1960 American Historical Association, Higham further developed his concept. He explained that the events of the post-war world had shattered the faith in progress that had underpinned the liberal political tradition and the historical vision of the Progressives. Where once change had been seen as both inevitable and welcome, it was now to be feared. Consequently, faith in mass democracy had been replaced with an appreciation of the stability of political institutions. The resultant historical temper was one that saw virtue in uniformity and agreement. Higham placed this conservative tendency within the wider movement of intellectuals towards reconciliation with American society. The desire to fit Hofstadter’s career into the narrative of the political journey of the post-war liberal intellectuals has inevitably led to his work being placed alongside those of whom Higham was critical. However, such an interpretation fails to consider Higham’s own qualification, that “the recognition of consensus in the past has not usually been unqualified. Nor has it always been presumed to sanction the status quo.”^20^

Whilst Higham was clear in his separation of Hofstadter from what he viewed as the pernicious influence of conservatism on American historiography, others were less discriminating. It was in responses to *The Age of Reform*, a work that Higham explicitly praised, that we first see Hofstadter’s work being placed within the consensus framework.^21^ The book was met with almost universal acclaim at the time of publication and Hofstadter was awarded his first Pulitzer Prize. However, William Appleman Williams’ assertion that Hofstadter had transformed history into ideology presaged the significant criticism that would follow in the decade after publication.^22^ The early years of the 1960s saw a coalescing of the reaction against consensus history and the antipathy towards those liberal intellectuals who were considered to have forsaken their radicalism for veneration of the American system. In many ways, Hofstadter was a victim of this reaction. To the younger historians, many of whom were politically involved and felt a sense of kinship with the reform movements of the past, Hofstadter’s critical assessment of Populism and Progressive was reflective of liberal intellectuals’ distaste for political radicalism. Norman Pollack was in little doubt about the reason for Hofstadter’s interpretation; his “basic methodological assumption is his consensus thesis.”^23^

The charge that Hofstadter’s study of the reform movements was indicative of the liberal intellectuals’ defence of the American political system was most clearly expressed in Michael P. Rogin’s *The Intellectuals and McCarthy*. Whilst ostensibly a study of McCarthyism, his primary concern was to call into question a political and historical point of view that he defined as “pluralism.” This viewpoint, consensus by another name, was markedly conservative, concerned with political stability and suspicious of mass movements. In inflating the threat of McCarthyism and imposing personal concerns upon their studies, historians like Hofstadter had been guilty of refracting “American history through the myopia of a traumatized intelligentsia.” Hofstadter’s reinterpretation of the reform movement was motivated by an overriding desire to venerate the “pluralist” system and to impugn its challengers. As such, it was the archetypal work of consensus history.^24^

Higham had distinguished between those who celebrated the supposed unity of the American past, and those who saw it as cause for concern. Nevertheless, the term consensus became inextricably linked to political conservatism. That his original essay, which had been written as a challenge to those who sought to impose a limiting framework upon history, became itself a fixed interpretation, was the cause of much regret for Higham. As the influence of the essay grew, he began to doubt its validity. By 1968, when he addressed a symposium to honour Merle Curti, he had rejected the idea that a consensus school existed. He had come to the realisation that his theory of consensus had been the result of his attempt to fit American historiography “into the interpretative framework my preconceptions had erected.”^25^ Yet Higham’s own admission that the concept itself had been an ideological construct had little impact on the essay’s influence. By the time Rogin published his work, the dominance of consensus history in the post-war years, the conservatism of its political underpinnings and Hofstadter’s key role in the movement were accepted facts. Furthermore, the interpretative model, coloured by a perception of Hofstadter’s later development had been extended backwards, its origins located in *The American Political Tradition*. It is this perception of Hofstadter and his work that continues to retain a hold. As Rick Perlstein, wrote recently in the New York Times, “Hofstadter was the leader of the “consensus” school of historians.”^26^ His definition of consensus as a historical illusion constructed for ideological purposes is evidence of the enduring influence of interpretations of Hofstadter formed in the 1960s.

Understandably, the association of his work with the consensus school was the source of great annoyance for Hofstadter. In a later edition of *The American Political Tradition*, he would bemoan the fact that his introduction had “become a first statement of a very controversial point of view…called consensus history.”^27^ Yet, at the time of publication, the book was overwhelmingly viewed as a trenchant critique of liberalism from the political left. As one reviewer noted, that “none of the major parties…questioned the immovable cornerstone of capitalist America” was a matter of the greatest regret to Hofstadter.^28^ Hofstadter’s radical outlook and distaste for capitalism were evident throughout the work. Indeed, when Higham himself addressed *The American Political Tradition* he commented that Hofstadter “wrote from a position so sympathetic to Beard and so critical of American business mores that his heresy seemed only a step to the left.”^29^

At the time of publication, not a single critic detected the roots of a nascent conservative historiographical movement within the book. Later interpretations have ignored the fact that he was intensely critical of the apparent lack of ideological struggle. The apparent consensus was a worrying discovery, rather than one that brought comfort. In fact, Hofstadter saw his book as an antidote to American self-celebration and an attempt to show its political heroes not as marmoreal saints but as “live and vulnerable figures of controversy.”^30^ The book’s primary function was to unravel the myths that had surrounded the nation’s leaders. An engagement with the book beyond the opening comments would provide ample evidence that Hofstadter’s concentration on consensus was intended to be descriptive rather than prescriptive.

Although some contemporary reviewers voiced concern at the disparity between the grand theoretical statement and the scope of the essays, only C. Vann Woodward foresaw the controversy that Hofstadter’s emphasis on the unity of cultural and political tradition might cause. Woodward wrote, “It is little wonder that such assumptions prompt a certain uneasiness (as they did in the mind of the reviewer), for in other hands they have contributed to the literature of nationalism and complacency.” However, as Woodward pointed out, “Not so in the hands of Mr Hofstadter.”^31^ If only later critics had been as discerning as Woodward, impressions of Hofstadter and *The American Political Tradition* might be very different.

Hofstadter had not intended to announce the coming of a new historical model, although he had come to the conclusion that the work of the Progressives could no longer function as convincing guides to the nation’s past. Their faith in nineteenth century ideas and institutions was no longer possible in the face of twentieth century realities. In this respect, his central concern was more political than historiographical. As he proclaimed in his introduction, “the traditional ground is shifting under our feet. It is imperative at this time of cultural crisis to gain fresh perspectives on the past.” He saw *The American Political Tradition* as a work of necessity, prompted by his concern with the “rudderless and demoralized state of American liberalism.” The introduction, rather than being a statement of celebration, was a call for a radical reconsideration of the liberal tradition. That the book was an attempt to commence the task of redefining and repositioning liberalism in light of the events of the first half of the twentieth century is one that has been lost. That this is so is a matter of great misfortune.^32^

## Footnotes

1 Caroline Sherman West to Richard Hofstadter, September 19, 1948, Richard Hofstadter Papers (RHP), Columbia Rare Books and Manuscript Library, Box 20.

2 John Higham, “The Cult of ‘American Consensus’: Homogenizing Our History,” *Commentary* 27 (February 1959), 94.

3 Draft introduction to *The American Political Tradition*, RHP, Box 3.

4 Richard Hofstadter to Harvey Swados, October 9, 1939, Harvey Swados Papers (HSP), Special Collections and University Archives, University of Massachusetts Amherst Libraries, Box 31.

5 Hofstadter to Swados, August 19, 1943, HSP, Box 31.

6 Ibid.

7 Draft introduction to *The American Political Tradition*.

8 C. Vann Woodward, “Review of The American Political Tradition,” *Mississippi Valley Historical Review*, 35 (1949), 682.

9 Hofstadter to Merle Curti, undated (early 1948), Merle Eugene Curti Papers, Wisconsin Historical Society.

10 Albert G. Huegli, “Review of American Political Tradition,” *The American Political Science Review*, 42:6 (December 1, 1948), 1215.

11 Hofstadter, *American Political Tradition*, 18, 19, 93, 94.

12 Ibid., 352.

13 Ibid., 36, 95, 97.

14 Ibid., 25, 228, 271, 342, 139.

15 Daniel Aaron, “Review: The American Political Tradition and the Men Who Made It,” *American Quarterly*, 1:1 (Spring 1949), 96.

16 Arthur Schlesinger, Jnr., “Review of The American Political Tradition,” The American Historical Review, 54:3, (April 1949), 613.

17 Hofstadter, Preface to Hebrew Edition, reprinted in *The American Political Tradition* (New York: Vintage, 1974); Arthur Schlesinger Jnr., “Review of *The American Political Tradition*,” 612.

18 Hofstadter, *American Political Tradition*, x.

19 Louis Hartz, The Liberal Tradition in America: An Interpretation of American Political Thought since the Revolution (New York: Harcourt Brace, 1955); Daniel Boorstin, The Genius of American Politics (Chicago: University of Chicago Press, 1953); John Higham, “The Cult of ‘American Consensus’”.

20 John Higham, “Beyond Consensus: The Historian as Moral Critic,” in *Writing American History: Essays on Modern Scholarship*, (Bloomington: Indiana University Press, 1970), 146. This caveat was not contained in the original version, published in *The American Historical Review*, 67:3, (April, 1962), 609–625.

21 Richard Hofstadter, *The Age of Reform: From Bryan to F.D.R.* (New York: Knopf, 1955).

22 William Appleman Williams, “The Age of Re-Forming History”, *Nation* (30 June, 1956), 552–554.

23 Norman Pollack, “Hofstadter on Populism: A Critique of The Age of Reform,” *Journal of Southern History* (November 1960), 479.

24 Michael P. Rogin, *The Intellectuals and McCarthy: The Radical Spec*ter (Cambridge, Mass.: M.I.T. Press, 1967), 2.

25 John Higham, “American Historiography in the 1960s,” in *Writing American History*, 159

26 Rick Perlstein, “I Thought I Understood the American Right. Trump Proved Me Wrong,” *New York Times*, April 11, 2017.

27 Hofstadter, *American Political Tradition*, xxvii.

28 Aaron, “Review: The American Political Tradition and the Men Who Made It,” 95.

29 John Higham, History: Professional Scholarship in America (New York: Harper Row, 1973), 213.

30 Hofstadter, *American Political Tradition*, xxxi.

31 Woodward, “Review of The American Political Tradition,” 681.

32 Hofstadter, *American Political Tradition*, x, vii.

